# Mental health problems and social supports in the COVID-19 healthcare workers: a Chinese explanatory study

**DOI:** 10.1186/s12888-020-02998-y

**Published:** 2021-01-12

**Authors:** Xue-Hui Fang, Li Wu, Lun-Shan Lu, Xiao-Hong Kan, Hua Wang, Yan-Jun Xiong, Dong-Chun Ma, Guo-Cui Wu

**Affiliations:** 1 Personnel Office, Anhui Provincial Chest Hospital (Anhui Institute of Tuberculosis Control), Hefei, 230022 Anhui China; 2 Anhui Provincial Chest Hospital affiliated to Anhui Medical University, Hefei, China; 3 School of Nursing, Anhui Medical University, Hefei, 230032 Anhui China; 4 Infection Hospital of Anhui Provincial Hospital, Hefei, 230001 Anhui China; 5 Science and Education Division, Anhui Provincial Chest Hospital (Anhui Institute of Tuberculosis Control), Hefei, Hefei, 230022 Anhui China; 6 Tuberculosis Department, Anhui Provincial Chest Hospital (Anhui Institute of Tuberculosis Control), Hefei, 230022 Anhui China; 7 Hospital Office, Anhui Provincial Chest Hospital (Anhui Institute of Tuberculosis Control), Hefei, 230022 China

**Keywords:** COVID-19, Tuberculosis, Social support, Lonely, Depression

## Abstract

**Background:**

Coronavirus disease 2019 (COVID-19) has spread rapidly in China and other overseas areas, which has aroused widespread concern. The sharp increase in the number of patients has led to great psychological pressure on health care workers. The purpose of this study was to understand their mental health status and needs, so as to provide a scientific basis for alleviating the psychological pressure of health care workers.

**Methods:**

Using a cross-sectional study design, 540 health care workers were randomly selected from two designated tuberculosis medical institutions in Anhui Province. The basic situation, perceived social support, depression level, loneliness and COVID-19 related knowledge were collected and analyzed by questionnaire.

**Results:**

A total of 511 valid questionnaires were finally retrieved. There were 139 people in epidemic prevention and control positions (27.20%). Depression level: People in isolation ward, fever clinic and pre-check triage were at the level of mild to moderate depression. Female was higher than male; nurse was higher than doctor; middle and junior job titles were higher than senior titles; junior college degree or below were higher than bachelor’s degree, master’s degree and above; isolation ward, fever clinic and pre-check triage were significantly higher than those of non-prevention and control positions (*p* < 0.05). Loneliness scores: Doctors were higher than that of medical technicians, and isolation ward, fever clinic and pre-check triage were higher than those of other medical departments (*p* < 0.05). Social support: Doctors were lower than that of medical technicians, and isolation ward, fever clinic and pre-check triage were significantly lower than those of other departments (*p* < 0.05). The score of social support was negatively correlated with depression and loneliness (*p* < 0.001), while depression was positively correlated with loneliness (*p* < 0.001). Health care workers most want to receive one-to-one psychological counseling (29.75%), and provide crisis management (24.07%). The awareness rate of health care workers on COVID-19’s knowledge was relatively high.

**Conclusions:**

The psychological problems of health care workers, especially women, nurses with low educational background, low professional title, and staff in the epidemic prevention and control positions are relatively serious.

## Background

The Coronavirus Disease 2019 (COVID-19) was first identified in Wuhan of Hubei Province, China in December, 2019. COVID-19 can result in severe and even fatal respiratory diseases such as acute respiratory distress syndrome [1]. It is similar to Severe Acute Respiratory Syndrome coronavirus (SARS-CoV) virus in its pathogenicity, clinical spectrum, and epidemiology [2]. As of May 12, 2020, the virus has infected more than 10 countries and regions around the world, with a total of 4,088,848 cases and 283,153 deaths [3]. Health care workers are at high risk of COVID-19 infection, a study by Wu et al. [4] showed that a total of 72,314 cases have been reported nationwide by February 11, 2020, of which 44,672 cases have been confirmed. Of all the confirmed cases, there were 1716 health workers (3.8%), of which 14.8% of the confirmed cases were diagnosed as severe or critical cases, and 5 cases died. Another study showed that [5] front-line health care workers are prone to psychological problems in public health emergencies. At present, the vaccine is still in the research stage, as the main force in the fight against the epidemic, lack of medical resources, health care workers are prone to all kinds of psychological problems in the face of high-intensity and high-risk work. Two recent studies in China both revealed that the incidence of anxiety and stress disorder is high among health care workers who were treating patients with COVID-19 infection during the epidemic period [6, 7]. The National Health Commission issued a public document requiring [8] that all localities should strengthen the psychological crisis intervention and guidance of health care workers.

The outbreak of COVID-19 has a negative impact on society and economy, leading to serious psychological problems [9, 10]. Countries such as India and Bangladesh have reported suicides [11–14], indicating an increase in suicidal tendency [15]. Fear of infection was the second largest factor leading to suicide [16]. Health care workers were faced with a high risk of disease exposure and worried about spreading the virus to family members, which may lead to psychological problems such as anxiety, depression and stress [17].

At present, some scholars have paid attention to the psychological status of health care workers during the epidemic, mainly focused on worry, anxiety and other psychological problem, there is still a lack of further research to deal with mental health problems. Social support can relieve individual psychological pressure, eliminate psychological obstacles and promote mental health. A study have proposed various forms of support, such as counseling service and peer support [18], to alleviate psychological problems. This study investigated the current situation of loneliness, depression and social support, and analyzed the mental health coping strategies, in order to improve the mental health level of medical staff and better carry out the fight against the epidemic situation. in order to improve the mental health level of health care workers and better carry out the work of fighting against the epidemic situation.

## Methods

### Participants

The method of random sampling was used to reduce the sampling error, and a questionnaire survey was conducted among health care workers from different positions from two provincial tuberculosis designated medical institutions in Anhui province, so that the survey results are more real and reliable. The questionnaire was distributed on the spot, the investigators checked the integrity of the questionnaire in time and followed it up, and took it back on the spot after filling it out, so as to avoid repetition and other possible negligence. The study subjects with a history of psychiatric illness were excluded.

### Measures

A demographic survey was conducted among the selected health care workers, including gender, occupation, job title, education level, marital status, epidemic prevention and control post, and Depressive state. Questionnaire was provided in the form of supplementary file (Questionnaire).

#### COVID-19 related knowledge

The following contents were investigated: Awareness rate of health care workers, including the source of infection, route of transmission, susceptible population and main clinical symptoms. In addition, an in-depth survey was conducted on mental health needs, such as favorite psychological demand and service items and expected counseling time.

#### Perceived social support scale (PSSS)

The scale was compiled in 1987 [19], revised by Chinese scholars and widely applied in China, Cronbach’s α = 0.88 [20]. It consists of 12 items, each of which is rated on a scale ranging from 1 to 7, and each item is randomly arranged, which is divided into three dimensions: family support, friend support and other support. The total support is the sum of the three dimensions, the higher the score, the higher the perceived social support [21]. Specific grading criteria: < 52 points: severe; 52–62: poor; 63–73: medium; 74–78: good; > 78: great [22].

#### Self-rating depression scale (SDS)

The scale includes 4 groups: psycho-emotional symptoms, somatic disorders, psychomotor disorders and psychological disorders of depression. The specific scoring method is as follows: each item is scored according to grades 1, 2, 3 and 4. The higher the score is, the more serious the degree of depression is, and the cumulative score was more than 40 points, which was judged as depression [23]. Specifically, 41–47: mild to moderate; 48–55: moderate to severe; ≥56: severe [24].

#### ULCA loneliness scale

The scale was compiled and revised by Russell et al. [25], including 11 “lonely” positive order items and nine “non-lonely” reverse order items, the items with asterisks should be in reverse order, and then each item should be added. The higher the score, the higher the degree of loneliness [26]. A total of 20–80 points, which were equally divided into three groups: low, medium and high [27].

### Statistical analyses

The data were double input by EpiData 3.1 and analyzed by SPSS 23.0. The quantitative data were expressed as Mean ± Standard Deviation (Mean ± SD), the categorical data were expressed as cases (percentage), *t*-test was used to compare the quantitative data between the two groups, one-way ANOVA was used to compare the quantitative data among different groups, Pearson correlation analysis was used when the variables were in accordance with bivariate normal distribution. The test level was α = 0.05.

### Ethics approval and consent to participate

Since this was an observational study without any interventions, which had no any adverse effects on the study subjects, thus only oral informed consent was obtained, the procedure had been approved by the Medical Ethics Committee of Anhui Chest Hospital (approval number: K2020–004).

## Results

### Demographic characteristics of health care workers in designated medical institutions for tuberculosis

A total of 540 questionnaires were distributed and the quality and integrity of the questionnaires were checked. Five hundred eleven eligible valid questionnaires were finally retrieved, with a response rate of 94.63%. The average age of 511 respondents was 31.19 ± 6.62, including 88 males (17.22%), 423 females (82.78%), the proportion of nurses was the highest (57.34%), and the proportion of bachelor degree was the highest (70.65%). Also, there were 139 people in epidemic prevention and control posts, accounting for 27.20%, of which fever clinic and pre-check triage accounted for the highest proportion (9.59%), and 186 health care workers had depression, accounting for 36.40% (Table 1).

**Table 1:** Demographic characteristics of health care workers [n (%)]

Characteristics	n (%)
Gender	
Male	88(17.22)
Female	423 (82.78)
Occupation	
Doctor	145 (28.38)
Nurse	293 (57.34)
Medical technology	73 (14.28)
Job title	
Junior	336 (65.75)
Intermediate	143 (27.99)
Senior	32 (6.26)
Education level	
Junior college and below	86 (16.83)
Bachelor’s degree	361 (70.65)
Master’s degree or above	64 (12.52)
Marital status	
Unmarried	167 (32.68)
Married	333 (65.17)
Divorced or widowed	11 (2.15)
Medical department	
Other medical department	372 (72.80)
Isolation ward	16 (3.13)
Fever clinic and pre-check triage	49 (9.59)
Imaging and laboratory diagnosis	43 (8.41)
Medicament	31 (6.07)
Depressive state	
Depression	186 (36.40)
Non-depression	325 (63.60)

### Comparison of social support, loneliness and depression scores among medical workers of different genders, occupations, job titles and educational levels

The perceived social support of health care workers was in the middle level, the medical technicians were relatively higher. The perceived sense of loneliness was low. There was no significant difference in the score of social support and loneliness among health care workers of different genders. The depression score of female was higher than that of male (*p* < 0.05). One-way ANOVA showed that there were significant differences in social support, loneliness and depression among different occupations. The scores of family support, friend support, other support and total support of doctors were significantly lower than those of medical technicians (all *p* < 0.05), the score of loneliness was higher than that of medical technicians (*p* < 0.05), and the depression score of nurses was higher than that of doctors (*p* < 0.05). The difference in the level of depression was statistically significant in terms of professional titles, the depression scores of intermediate and junior health care workers were significantly higher than those of senior titles (all *p* < 0.05). Healthcare workers with educational attainment of junior college and below were higher than those with bachelor’s degree or master’s degree or above in the scores of depression (all *p* < 0.05) (Table 2).

**Table 2:** Comparison of different genders, occupations, job titles and educational levels (Mean ± SD)

Characteristics	Family support	Friends support	Other support	Total support	Loneliness	Depression
Gender						
Male	23.84 ± 3.59	23.20 ± 3.63	22.77 ± 3.81	69.82 ± 10.23	35.23 ± 11.20	34.27 ± 10.63
Female	23.91 ± 3.50	23.01 ± 3.59	22.75 ± 3.60	69.67 ± 9.83	37.28 ± 10.37	36.97 ± 9.55
*t*	0.17	0.47	0.04	0.13	1.67	2.37
*P*	0.862	0.639	0.97	0.901	0.096	0.018^****^
Occupation						
Doctor	23.60 ± 3.50	22.83 ± 3.29	22.51 ± 3.58	68.94 ± 9.39	36.87 ± 11.26	35.46 ± 10.59
Nurse	23.74 ± 3.61	22.81 ± 3.78	22.54 ± 3.63	69.09 ± 10.19	37.73 ± 10.40	37.59 ± 9.73^*^
Medical technology	25.15 ± 2.82^*^	24.40 ± 3.11^*^	24.11 ± 3.47^*^	73.66 ± 8.77^*^	33.84 ± 9.07^*^	34.26 ± 7.63
*F*	5.58	6.20	6.05	7.00	4.04	4.60
*P*	0.004^****^	0.002^****^	0.003^****^	0.001^****^	0.018^****^	0.01^****^
Job title						
Junior	23.93 ± 3.51	23.15 ± 3.64	22.93 ± 3.53	70.03 ± 10.00	36.97 ± 10.64	36.74 ± 9.71^**^
Intermediate	23.64 ± 3.63	22.63 ± 3.60	22.33 ± 3.62	68.60 ± 9.83	37.33 ± 10.21	37.13 ± 10.00^**^
Senior	24.56 ± 2.93	23.72 ± 2.83	22.81 ± 4.50	71.09 ± 8.83	34.72 ± 10.96	31.38 ± 8.34
*F*	0.98	1.67	1.41	1.39	0.81	4.85
*P*	0.375	0.189	0.246	0.249	0.446	0.008^****^
Education level						
Junior college or below	23.31 ± 3.65	22.73 ± 3.59	22.26 ± 3.67	68.30 ± 10.04	37.92 ± 10.18	39.02 ± 9.69
Bachelor’s degree	24.02 ± 3.41	23.06 ± 3.61	22.78 ± 3.66	69.86 ± 9.79	36.75 ± 10.70	36.24 ± 9.59^***^
Master’s degree or above	24.00 ± 3.83	23.36 ± 3.54	23.30 ± 3.31	70.66 ± 10.19	36.63 ± 10.19	34.67 ± 10.50^***^
*F*	1.45	0.57	1.54	1.21	0.46	4.16
*P*	0.236	0.565	0.215	0.300	0.633	0.016^****^

*Note.* ^*^: Compared with doctors, *p* < 0.05; ^**^: Compared with senior job titles, *p* < 0.05; ^***^: Compared with junior college or below, *p* < 0.05; ^****^: The result of *t*-test or one-way ANOVA, *p* < 0.05

### Comparison of social support, loneliness and depression scores among health care workers of different positions

The total support of various medical departments was in the middle level, and the level of loneliness was low. The health care workers in isolation ward, fever clinic and pre-check triage were at the level of mild to moderate depression. There was no significant difference in the score of total social support between the health care workers in COVID-19 prevention and control posts and those in other medical department but the scores of loneliness and depression of health care workers in prevention and control posts were significantly higher than those in other medical department (*p* < 0.05). One-way ANOVA showed that there were significant differences in the scores of social support, loneliness and depression in specific prevention and control posts. Among them, the score of social support of health care workers in isolation ward, fever clinic and pre-check triage were lower than those in other medical department (*p* < 0.05), while the scores of loneliness and depression were significantly higher than those in other department (*p* < 0.05), and the scores of depression even reached the diagnostic criteria. The social support of health care workers in imaging and laboratory diagnosis positions was higher than that of other department (*p* < 0.05). There was no significant difference between Medicament and other medical department in terms of social support, loneliness and depression (Table 3).

**Table 3:** Comparison among health care workers of different departments (Mean ± SD)

Characteristics	Family support	Friends support	Other support	Total support	Loneliness	Depression
Department						
Other medical department	24.00 ± 3.33	23.12 ± 3.33	22.72 ± 3.52	69.84 ± 9.27	36.24 ± 10.09	35.47 ± 9.25
Prevention and control post	23.64 ± 3.95	22.83 ± 4.22	22.86 ± 3.90	69.32 ± 11.40	38.78 ± 11.50	39.29 ± 10.62
*t*	0.95	0.74	0.38	0.48	2.44	3.99
*P*	0.345	0.461	0.707	0.634	0.015^**^	<0.001
Medical department						
Other medical department	24.00 ± 3.41	23.12 ± 3.33	22.72 ± 3.52	69.84 ± 9.27	36.24 ± 10.09	35.47 ± 9.25
Isolation ward	21.13 ± 5.76^*^	20.56 ± 5.20^*^	20.81 ± 5.12^*^	62.50 ± 15.56^*^	46.81 ± 11.91^*^	45.00 ± 11.06^*^
Fever clinic and pre-check triage	22.59 ± 3.96^*^	21.27 ± 4.74^*^	21.41 ± 3.87^*^	65.27 ± 11.70^*^	43.57 ± 10.99^*^	44.55 ± 11.17^*^
Imaging and laboratory diagnosis	24.98 ± 3.41	24.44 ± 3.38^*^	24.49 ± 3.55^*^	73.91 ± 9.61^*^	34.05 ± 10.48	35.33 ± 8.62
Medicament	24.74 ± 2.19	24.23 ± 2.00	23 .94 ± 2.13	72.90 ± 5.83	33.65 ± 7.66	33.55 ± 5.89
*F*	5.96	7.82	6.39	7.75	11.17	14.63
*P*	<0.001	<0.001	<0.001	<0.001	<0.001	<0.001

*Note.* ^*^: Compared with other medical department, *p* < 0.05, ^**^: The result of *t*-test, *p* < 0.05

### Correlation analysis of social support with depression and loneliness

Family support, friend support, other support and total social support of health care workers were negatively correlated with the scores of depression and loneliness (*p* < 0.001). Also, there was a significant positive correlation between the score of depression and the score of loneliness (*p* < 0.001) (Table 4).

**Table 4:** Correlation analysis of social support with depression and loneliness

Variables	Depression		Loneliness	
	***r***	***p***	***r***	***p***
Family support	−0.51	<0.001	−0.56	<0.001
Friends support	−0.49	<0.001	−0.58	<0.001
Other support	− 0.47	<0.001	− 0.57	<0.001
Total support	−0.53	<0.001	−0.62	<0.001
Depression	1	–	0.75	<0.001

### The need for mental health and awareness rate of COVID-19 related knowledge among health care workers

The top three psychological needs that health care workers were willing to accept were one-to-one psychological counseling (29.75%), psychological lectures (27.20%) and participating in interactive groups (18.59%). The top three psychological services needed were crisis event management (24.07%), emotional management (21.33%) and stress and frustration coping (21.13%). Most health care workers were willing to have psychological counseling during normal working hours (61.84%). The awareness rate of COVID-19 related knowledge was relatively high, the highest was the source of infection (100%), and the lowest was the clinical manifestation (93.54%). It shows that the understanding of COVID-19 related clinical manifestations among health care workers remains to be deepened (Table 5).

**Table 5:** The need for mental health and awareness rate of COVID-19 [n (%)]

Characteristics	n (%)	Characteristics	n (%)
Psychological demand		Consultation time	
One-to-one consultation	152 (29.75)	working period	316 (61.84)
Psychological lecture	139 (27.20)	weekend	76 (14.87)
Interactive group	95 (18.59)	lunch break	22 (4.31)
Network Consultation	90 (17.61)	Other time	97 (18.98)
Telephone Consultation	35 (6.85)	Awareness rate	
Service items		Infectious source	511 (100.00)
Crisis event management	123 (24.07)	Pathogen	510 (99.80)
Emotional management	109 (21.33)	Transmission route	510 (99.80)
Stress and frustration coping	108 (21.13)	Susceptible population	489 (95.69)
Self-awareness and acceptance	78 (15.26)	Clinical manifestation	478 (93.54)
Interpersonal communication	59 (11.55)		
Parent-child relationship	34 (6.65)		

## Discussion

COVID-19 has been listed as Class B infectious disease according to the Law of the People’s Republic of China on Prevention and Control of Infectious Diseases in China, and it was managed according to Class A infectious diseases, which is the strictest way of prevention and control management, and there are differences in prevention and control measures. When medical institution discovers a Class A infectious disease, it shall promptly take the following measures: (1) patients and pathogen carriers shall be isolated and treated, and the period of isolation shall be determined according to the results of medical examination; (2) for suspected patients, separate isolation treatment in a designated place before diagnosis. (3) patients, pathogen carriers and close contacts of suspected patients in medical institutions shall conduct medical observation and take other necessary preventive measures at designated places. When medical institution discovers a patient with a Class B infectious disease, it shall take necessary measures to treat and control the spread of the disease according to its condition. Disinfection and harmless disposal shall be carried out in accordance with the provisions of laws and regulations. As an emerging infectious disease, much is still unknown about how the COVID-19 spreads, so it is easy to cause panic and psychological problems. A population-based cross-sectional study [28] explored the psychological reaction of people in China in the early stages of the COVID-19 outbreak, and found that the rates of moderate and severe anxiety among volunteers fighting the epidemic were 32.7 and 20.4% in Wuhan and Shanghai, respectively, suggesting that during the rising stage of the outbreak, the physical and mental reactions of the masses were significant.

There has been an increase in studies reporting a high incidence of depression among health care workers in Asia, the prevalence of depressive symptoms was 20.1% [29] even 50.4% [30], and calling on them to undergo screening and counseling [18]. Studies have shown that 36.4% of health care workers had depression symptoms and there was a significant positive correlation between depression and loneliness, which was higher than that reported in a previous study by Qi et al. [31], and the depression scores of health care workers with lower job title and educational background were higher. The loneliness of health care workers was generally at a low level. The score of loneliness of doctors was higher than that of medical technicians. The health care workers in isolation ward, fever clinic and pre-check triage had mild to moderate depression, and the scores of depression and loneliness were higher than those of other medical departments. It showed that the psychological problems of those with low job titles and academic qualifications, as well as those in isolation ward, fever clinic and pre-check triage were relatively serious, and more attention should be paid to the psychological problems of them. Shao et al. [32] found that during the period of fighting against SARS, the mental health level of health care workers was lower than the domestic norm, and there were mental health problems such as anxiety, fear, etc. And health care workers in prevention and control posts such as isolation wards and fever clinics, rushed to the front line of the epidemic. Problems encountered in clinical work could not be solved in time, and there was no psychological comfort. In particular, some doctors were in a state of isolation for a long time and cannot be reunited with their families [17], but also worried about the risk of family infection, making them feel lonely and helpless, extremely prone to psychological problems.

In addition, this study found that depression had differences in gender, occupation, professional title, education level and different positions. Among them, the depression score of nurses was higher than that of doctors. Qi et al. [31] found that clinical nurses have psychological problems in the special period of facing COVID-19, another study by Jiao et al. [33] shows that in the fight against the epidemic, nurses have a high intensity of tasks, and they have a high probability of contact with infected patients. Moreover, the drugs need to be verified by further clinical trials [34]. Therefore, in the face of many uncertainties, clinical nurses are prone to varying degrees of psychological problems.

This study suggests that the social support of health care workers was in the middle level and negatively correlated with the scores of depression and loneliness. Further analysis of the social support of health care workers is particularly important for a comprehensive understanding of mental health status. Among them, the scores of social support of health care workers in prevention and control positions were lower. The reason is that they are more likely to come into contact with COVID-19 patients, and their family and friends may be worried about being infected, resulting in relatively low social support. Mental health factors may lead to suicide [13]. In the absence of social support, health care workers tend to choose negative coping styles, such as self-attack and retreat, thus affecting their mental health [35]. Therefore, close attention should be paid to improve the psychological coping ability of health care workers under stress [10, 36], in order to enhance their mental health level, and then carry out epidemic prevention and control work more effectively.

In the face of the psychological problems that need to be solved urgently, this study continues to investigate the psychological needs of health care workers, in order to provide a better solution for hospital management and improve mental health. A study proposed forms of psychological support such as telemedicine and informal support groups [37], and the following reference improvement measures were obtained from this study, such as mental health lectures, psychological counseling and psychological guidance for them, because healthy psychological counseling can maintain a positive and stable state of mind to deal with unexpected situations and reduce the risk of mental disorders such as anxiety and depression [38]. In addition, it is also very important to actively care for the families of health care workers and make them feel at ease with their work. It is suggested that managers should communicate more, understand their difficulties encountered in work and life, give timely help, and reduce their life stress [39].

Mental health is not only related to individual health, but also affects social function and professional ability [40]. During the epidemic period of COVID-19, special attention should be paid to it. Health care workers, as front-line personnel in the fight against the epidemic, face both physical and mental pressure. The quality of their work is not only related to the life safety of patients, but also related to whether the epidemic can be effectively controlled and social stability. Therefore, we should actively take mental health intervention measures for health care workers according to the psychological needs of them. This study points out the health care workers who need to pay attention to during the epidemic, and provides reference for the prevention and management of public health emergencies.

## Study limitations

This study has some limitations. First of all, despite the widespread spread of COVID-19, this study only investigates the mental health status of health care workers in two designated tuberculosis medical institutions during the epidemic period. The conclusions are limited, so multicenter studies should be considered. Secondly, the mental health status is dynamic, and the results of cross-sectional survey can only reflect the psychological information at a certain point in time. The design method of longitudinal study will make the research results more rigorous. Finally, the baseline depression status and socioeconomic status variation of study subjects were not investigated, which may be potential confounding factors.

## Conclusion

During the epidemic period of COVID-19, the psychological problems of health care workers, especially women, nurses with low educational background, low professional title, and staff in the epidemic prevention and control positions are relatively serious, and the problem of loneliness was relatively mild, mainly in terms of depression. Targeted intervention measures should be taken according to their psychological needs to give more support and improve their mental health.

## Data Availability

The datasets used in the current study are available from the corresponding author on reasonable request.

## Abbreviations

COVID-19: Coronavirus disease 2019
PSSS: Perceived Social support scale
SDS: Self-rating Depression scale

## References

[1] Chen N, Zhou M, Dong X, Qu J, Gong F, Han Y (2020). Epidemiological and clinical characteristics of 99 cases of 2019 novel coronavirus pneumonia in Wuhan, China: a descriptive study. LANCET.

[2] Kannan S, Shaik SAP, Sheeza A, Hemalatha K (2020). COVID-19 (novel coronavirus 2019) - recent trends. Eur Rev Med Pharmacol Sci.

[3] Organization WH (2020). Coronavirus disease (COVID-2019) situation report – 113.

[4] Wu Z, McGoogan JM (2020). Characteristics of and important lessons from the coronavirus disease 2019 (COVID-19) outbreak in China: summary of a report of 72314 cases from the Chinese Center for Disease Control and Prevention. JAMA.

[5] Zuo DM, Chen Y, Zhu NC, Xu AP, Shi CQ (2007). Investigation and analysis of anxiety and depression of front-line medical staff in a department during public health emergencies. Armed Police Med.

[6] Huang JZ, Han MF, Luo TD, Ren AK, Zhou XP (2020). Mental health survey of 230 medical staff in a tertiary infectious disease hospital for COVID-19. Zhonghua Lao Dong Wei Sheng Zhi Ye Bing Za Zhi.

[7] Xiao H, Zhang Y, Kong D, Li S, Yang N (2020). The effects of social support on sleep quality of medical staff treating patients with coronavirus disease 2019 (COVID-19) in January and February 2020 in China. Med Sci Monit.

[8] Commission NH (2020). Circular of the National Health Commission on implementing measures to improve the working conditions of front-line medical personnel and pay practical attention to the physical and mental health of medical personnel.

[9] Islam SMD, Bodrud-Doza M, Khan RM, Haque MA, Mamun MA (2020). Exploring COVID-19 stress and its factors in Bangladesh: a perception-based study. Heliyon.

[10] Mamun MA, Griffiths MD (2020). First COVID-19 suicide case in Bangladesh due to fear of COVID-19 and xenophobia: possible suicide prevention strategies. Asian J Psychiatr.

[11] Dsouza DD, Quadros S, Hyderabadwala ZJ, Mamun MA (2020). Aggregated COVID-19 suicide incidences in India: fear of COVID-19 infection is the prominent causative factor. Psychiatry Res.

[12] Bhuiyan AKMI, Sakib N, Pakpour AH, Griffiths MD, Mamun MA (2020). COVID-19-related suicides in Bangladesh due to lockdown and economic factors: case study evidence from media reports. Int J Ment Health Ad.

[13] Mamun MA, Bodrud-Doza M, Griffiths MD (2020). Hospital suicide due to non-treatment by healthcare staff fearing COVID-19 infection in Bangladesh?. Asian J Psychiatr.

[14] Mamun MA, Chandrima RM, Griffiths MD. Mother and son suicide pact due to COVID-19-related online learning issues in Bangladesh: an unusual case report. Int J Ment Health Ad. 2020. 10.1007/s11469-020-00362-5 PMC7340761 32837439

[15] Griffiths MD, Mamun MA (2020). COVID-19 suicidal behavior among couples and suicide pacts: case study evidence from press reports. Psychiatry Res.

[16] Mamun MA, Ullah I (2020). COVID-19 suicides in Pakistan, dying off not COVID-19 fear but poverty? – the forthcoming economic challenges for a developing country. Brain Behav Immun.

[17] Usman N, Mamun M, Ullah I (2020). COVID-19 infection risk in pakistani health-care workers: the cost-effective safety measures for developing countries. Soc Health Behavior.

[18] Ng QX, De Deyn M, Lim DY, Chan HW, Yeo WS (2020). The wounded healer: a narrative review of the mental health effects of the COVID-19 pandemic on healthcare workers. Asian J Psychiatr.

[19] Blumenthal JA, Burg MM, Barefoot J, Williams RB, Haney T, Zimet G (1987). Social support, type a behavior, and coronary artery disease. Psychosom Med.

[20] Zhu X, Ming Y, Liu J, Liu L, Cheng K, Mao P (2020). Sleep quality and psychosocial factors in liver transplant recipients at an outpatient follow-up Clinic in China. Ann Transpl.

[21] Wang XD, Wang XL, Ma H (1999). Manual of mental health rating scale (updated). Beijing: China Mental Health Magazine.

[22] Xueqin H. Study on the relationship between the health literacy, perceived social support and quality of life of patients with coronary heart disease: Nanchang University; 2018.

[23] Inoue N, Fukuyama K, Hirayama S, Yoshioka T, Ozawa T, Iwata S (2016). Cardiovascular risk assessment using LOX-index and self-rating depression scale. IJC Metab Endoc.

[24] Dunstan DA, Scott N, Todd AK (2017). Screening for anxiety and depression: reassessing the utility of the Zung scales. Bmc Psychiatry.

[25] Russell D, Peplau LA, Ferguson ML (1978). Developing a measure of loneliness. J Pers Assess.

[26] Zhou T, Shang Z, Wang D. Emotion suppression in multiple social contexts and its effects on psychosocial functioning: an investigation with Chinese samples. Asian J Soc Psychol. 2016;19(4):311.

[27] Hai H. A study on the current situation of college students’ loneliness and its influencing factors: Jiangxi normal University; 2004.

[28] Qian MC, Wu QH, Wu P, Hou ZY, Liang YX, Cowling BJ, et al. Psychological responses, behavioral changes and public perceptions during the early phase of the COVID-19 outbreak in China: a population based cross-sectional survey. medRxiv. 2020. 10.1136/bmjopen-2020-040910 PMC7545627 33033099

[29] Huang Y, Zhao N (2020). Generalized anxiety disorder, depressive symptoms and sleep quality during COVID-19 outbreak in China: a web-based cross-sectional survey. Psychiatry Res.

[30] Lai J, Ma S, Wang Y, Cai Z, Hu J, Wei N (2020). Factors associated with mental health outcomes among health care workers exposed to coronavirus disease 2019. JAMA Netw Open.

[31] Qi JJ, Liu LM, Li WT, Xu YH, Shan X (2020). Investigation and analysis on the psychological status of clinical nurses facing novel coronavirus pneumonia. Gen Pract Nurs.

[32] Shao L, Xiao SH, Cao TY, Xia J, Li XD (2004). Investigation and analysis of mental health status of young medical staff in hospital during the period of SARS. North China Natl Defense Med.

[33] Jiao LN, Cui JZ, Zhou AR, Sun J, Shan L (2017). Effect of ABC emotional management training method on anxiety and depression of nurses. Chinese Disaster Rescue Med.

[34] Yang XY, Miao CL, Jin MD, Zhou DD, Zhuang JQ, Hong J (2020). Research status and progress of COVID-19 in 2019. Chinese Critical Care Emerg Med.

[35] Zhang RM (2012). Study on the relationship between mental health status, coping style and social support of medical staff. China Health Nutr (late issue).

[36] Liang ZQ, Xue YZ, Zhang KR, Shi XY, Jiang F (2004). Study on coping style of medical staff under stress. China Public Health.

[37] Ng QX, Chee KT, De Deyn M, Chua Z (2020). Staying connected during the COVID-19 pandemic. Int J Soc Psychiatry.

[38] Ding L, Sun GS, Zhang YQ, Zhu HZ, Zhang HQ, Ye NY (2019). Analysis of the correlation between work stress, coping style and anxiety and depression of nurses in emergency department. Nurs Pract Res.

[39] Chu WJ (2017). Investigation and intervention suggestions on anxiety and depression of nurses in intensive care unit. J Tradit Chinese Med Manag.

[40] Yuan J, Shang CH, Zhang M, Xin G, Pang JJ, Wang J (2017). The influencing factors of anxiety and depression of medical staff in 4 hospitals in Tangshan in 2016 and their relationship with psychological elasticity. Occup Health.

